# Impact of pre-stroke dependency on outcome after endovascular therapy in acute ischemic stroke

**DOI:** 10.1007/s00415-020-10172-3

**Published:** 2020-08-31

**Authors:** Lisa Oesch, Marcel Arnold, Corrado Bernasconi, Johannes Kaesmacher, Urs Fischer, Pascal J. Mosimann, Simon Jung, Thomas Meinel, Martina Goeldlin, Mirjam Heldner, Bastian Volbers, Jan Gralla, Hakan Sarikaya

**Affiliations:** 1 Department of Neurology, Bern University Hospital, Freiburgstrasse 10, 3010 Bern, Switzerland; 2 Department of Diagnostic and Interventional Neuroradiology, Bern University Hospital, Freiburgstrasse 10, 3010 Bern, Switzerland; 3 Department of Diagnostic, Interventional and Pediatric Radiology, Bern University Hospital, Freiburgstrasse 10, 3010 Bern, Switzerland; 4 Department of Neurology, University of Erlangen-Nuremberg, Erlangen, Germany

**Keywords:** Ischemic stroke, Endovascular treatment, Dependency, Disability, Outcome

## Abstract

**Background and purpose:**

Current demographic changes indicate that more people will be care-dependent due to increasing life expectancy. Little is known about impact of preexisting dependency on stroke outcome after endovascular treatment (EVT).

**Methods:**

We compared prospectively collected baseline and outcome data of previously dependent vs. independent stroke patients (prestroke modified Rankin Scale score of 3–5 vs. 0–2) treated with EVT. Outcome measures were favorable 3-month outcome (mRS ≤ 3 for previously dependent and mRS ≤ 2 for independent patients, respectively), death and symptomatic intracranial hemorrhage (sICH).

**Results:**

Among 1247 patients, 84 (6.7%) were dependent before stroke. They were older (81 vs. 72 years of age), more often female (61.9% vs. 46%), had a higher stroke severity at baseline (NIHSS 18 vs. 15 points), more often history of previous stroke (32.9% vs. 9.1%) and more vascular risk factors than independent patients. Favorable outcome and mortality were to the disadvantage of independent patients (26.2% vs. 44.4% and 46.4% vs. 25.5%, respectively), whereas sICH was comparable in both cohorts (4.9% vs. 5%). However, preexisting dependency was not associated with clinical outcome and mortality after adjusting for outcome predictors (OR 1.076, 95% CI 0.612–1.891; *p* = 0.799 and OR 1.267, 95% CI 0.758–2.119; *p* = 0.367, respectively).

**Conclusion:**

Our study underscores the need for careful selection of care-dependent stroke patients when considering EVT, given a less favorable outcome observed in this cohort. Nonetheless, EVT should not systematically be withheld in patients with preexisting disability, since prior dependency does not significantly influence outcome.

## Background and purpose

Current demographic changes indicate that an increasing number of people will need external help in their daily life, given the rising life expectancy and incidence of care dependency among older people in Western countries [1]. As an example, an estimated 3.4 million people will be care-dependent in Germany by 2030 [2]. Concurrently, stroke has reached epidemic proportions worldwide [3] and endovascular treatment (EVT) is an approved and preferred intervention to achieve reperfusion in large cerebral artery occlusions [4, 5]. However, little is known about the impact of preexisting dependency on stroke outcome treated with EVT as patients with modified Rankin Scale (mRS) score ≥ 2 were excluded from latest randomized trials [5]. Thus, current guidelines recommend a prestroke mRS score 0–1 for patients being considered for EVT [6, 7]. We aimed to assess the clinical outcomes in care-dependent stroke patients treated with EVT.

## Patients and methods

This study was based on the Bernese stroke center database, a systematic prospective registry of consecutive patients with ischemic stroke treated at the Stroke Center of University Hospital of Berne, Switzerland. It was approved by the Local Ethics Committee Bern. For this study, we analyzed all stroke patients who underwent EVT (mechanical thrombectomy and/or intraarterial thrombolysis (IAT) with urokinase) between January 2005 and December 2016. Requests for access to the dataset from qualified researchers trained in human subject confidentiality protocols may be sent to Department of Neurology, University Hospital Berne (marcel.arnold@insel.ch). The following variables were prospectively collected as defined previously [8–10]: age, sex, prestroke mRS score, arterial hypertension, diabetes mellitus, hyperlipidemia, smoking status, history of coronary artery disease and previous stroke, antithrombotic medication at stroke onset, stroke onset-to-treatment time and stroke etiology according to the Trial of ORG 10172 in Acute Stroke Treatment (TOAST) criteria. Clinical stroke severity was assessed by a stroke neurologist at admission using the National Institutes of Health Stroke Scale (NIHSS) score [11]. Preexisting dependency was defined as prestroke mRS score 3–5, whereas patients with a prestroke mRS score 0–2 were classified as independent. We performed EVT according to our institutional guidelines as described before [12]. According to our local practice, prestroke dependency was not a strict exclusion criterion for EVT in acute ischemic stroke as we assumed that dependent patients with large vessel occlusion may also benefit from EVT. However, final treatment decision was individualized on a case-by-case basis at the discretion of the interdisciplinary team of neurologists and neuroradiologists. Patients were treated with intra-arterial urokinase, mechanical interventions, or both. Patients within 4.5 h after symptom onset were additionally treated with intravenous thrombolysis (IVT) [7]. All patients treated with EVT were admitted to intermediate or intensive care unit for at least 24 h. Brain imaging with MRI or CT was systematically performed 24 h after intervention and in any case of clinical deterioration. Symptomatic intracranial hemorrhage (sICH) was defined according to ECASS II criteria [13]. Primary outcome measures were as follows: (1) favorable clinical outcome at 3 months (mRS score ≤ 2 in independent patients and mRS score ≤ 3 in dependent patients), (2) death within 3 months and (3) occurrence of sICH. The endpoints were prospectively assessed during hospital stay and at 3-month outpatient visits.

## Statistical analysis

We compared demographic and baseline characteristics between prestroke dependent and independent patients using Fisher exact test for dichotomous variables and Wilcoxon rank-sum test for continuous variables in univariate analyses. The independent effect of prestroke dependency on endpoints was assessed in a multivariable logistic regression model. Any variable with p < 0.1 in the univariate analysis was entered into the regression model. Age and baseline NIHSS score were entered as mandatory into the model because they have been proven to be independent predictors of clinical outcome after stroke [14]. All tests were two-sided and the level of statistical significance was set to 0.05. Statistical analyses were performed using the statistical software R (version 3.1.2; R Core Team [2014]; R: A Language and Environment for Statistical Computing; R Foundation for Statistical Computing, Vienna, Austria).

## Results

A total of 1247 patients were eligible for this study. Of these, 84 (6.7%) were previously dependent. Baseline characteristics of both groups are detailed in Table 1. When compared with independent patients, those with prestroke dependency were older (81 vs. 72 years; *p* < 0.0001), more often female (61.9% vs. 46%; *p* = 0.0063), suffered more often from diabetes mellitus (31% vs. 15.9%; *p* = 0.0013) and arterial hypertension (79.8% vs. 67.6%; *p* = 0.0207). Furthermore, dependent patients had more often a history of previous stroke (32.9% vs. 9.1%;* p* < 0.0001) and pretreatment with antithrombotics (65.1% vs. 42.6%; *p* = 0.0001). Baseline NIHSS score was slightly higher in dependent patients (18 vs. 15 points; *p* = 0.0113), whereas stroke etiology and time from stroke onset to EVT did not significantly differ between both groups.

**Table 1:** Baseline characteristics according to prestroke dependency status in patients treated with endovascular treatment

	Prestroke dependent (mRS 3–5)	Prestroke independent (mRS 0–2)	Dependent vs independent
	Value	Value	*p* value
Age, y, median (IQR)	81 (73.75–85)	72 (60–79)	< 0.0001
Female sex (%)	52/84 (61.9)	535/1163 (46.0)	0.0063
Baseline NIHSS score, median (IQR)	18 (11–21)	15 (10–19)	0.0113
Arterial hypertension (%)	67/84 (79.8)	784/1160 (67.6)	0.0207
Diabetes mellitus (%)	26/84 (31.0)	185/1161 (15.9)	0.0013
Hyperlipidemia (%)	43/82 (52.4)	661/1147 (57.6)	0.3583
Smoking (%)	9/64 (14.1)	237/1063 (22.3)	0.1595
Coronary Artery Disease (%)	14/82 (17.1)	217/1154 (18.8)	0.771
History of previous stroke (%)	27/82 (32.9)	105/1158 (9.1)	< 0.0001
Pre-stroke use of antithrombotics (%)	54/83 (65.1)	491/1153 (42.6)	0.0001
OTT [min], median (IQR)	276.5 (199.5–352.25)	272 (209–363)	0.5836
Cause of stroke			0.1325
Large artery atherosclerosis (%)	5/84 (6)	170/1163 (14.6)	
Cardiac embolism (%)	41/84 (48.8)	485/1163 (41.7)	
Small artery disease (%)	0/84 (0)	1/1163 (0.1)	
Other determined cause (%)	3/84 (3.6)	69/1163 (5.9)	
Undetermined cause (%)	35/84 (41.7)	438/1163 (37.7)	

*IQR* indicates Interquartile Range; *NIHSS* National Institutes of Health Stroke Scale; *OTT* onset to treatment time

Clinical outcomes are summarized in Table 2. At 3 months, dependent patients less often reached favorable outcome (26.2% vs. 44.4%; *p* = 0.0013) and had higher mortality rates (46.4% vs. 25.5%; *p* < 0.0001) than independent patients, whereas sICH did not significantly differ between the groups (4.9% vs. 5%; *p* = 1.000).

**Table 2:** Outcome according to prestroke dependency status in patients treated with endovascular treatment

	Outcome measures			
	Prestroke dependent [*n*/*N* (%)]	Prestroke independent [*n*/*N* (%)]	*p* value Unadjusted [OR, 95% CI]	*p* value Adjusted* [OR, 95% CI]
Death at 3 months	39/84 (46.4)	297/1163 (25.5)	< 0.0001	0.367*
			[2.525 (1.567 – 4.049)]	[1.267 (0.758 – 2.119)]
Good outcome at 3 months**	22/84 (26.2)	516/1163 (44.4)	0.0013	0.799*
			[0.445 (0.257 – 0.746)]	[1.076 (0.612 – 1.891)]
sICH	4/81 (4.9)	57/1133 (5)	1	0.833
			[0.980 (0.252 – 2.755)]	[1.124 (0.380 – 3.324)]

*OR* indicates odds ratio; *CI* confidence interval; *sICH* symptomatic intracranial hemorrhage; *NIHSS* National Institutes of Health Stroke Scale

^*^Adjusted for age, sex, dependency status, baseline NIHSS score, arterial hypertension, diabetes mellitus, pre-stroke use of antithrombotics, and history of previous stroke **Good outcome was defined as 3-month modified Rankin scale (mRS) score 0–3 for previously care-dependent patients and mRS score 0–2 for previously independent patients

For multivariable regression analyses, the following covariates were entered into the model: age, sex, dependency status, baseline NIHSS score, arterial hypertension, diabetes mellitus, pre-stroke use of antithrombotics, and history of previous stroke. After adjusting for these covariates, dependency status was no more associated with favorable outcome (odds ratio [OR], 1.076; 95% confidence interval [CI], 0.612–1.891; *p* = 0.799), mortality (OR, 1.267; 95% CI 0.758–2.119; *p* = 0.367), or sICH (OR, 1.124 95% CI 0.380–3.324; *p* = 0.833).

## Discussion

This study examined the impact of pre-existing dependency on stroke outcome after EVT and revealed some important findings. First, the rate of care-dependent patients undergoing EVT in our cohort was similar as compared with a large multicenter study investigating intravenous thrombolysis (IVT) in 7430 stroke patients (6.7% vs. 6.6%) [15]. This rate is also similar to the proportions reported in large trials of SITS-EAST and SITS-MOST, meaning that the willingness to thrombolyse care-dependent stroke patients has not increased [16, 17]. Thus, care-dependent patients constitute still a minor but existent subgroup for stroke physicians in their daily clinical work [18]. However, their proportion may markedly increase in near future with respect to the recent demographic changes, which again underlines the role of this study [19]. In line with this, the proportion of care-dependent stroke patients undergoing treatment with EVT was higher (11%) according to a recently published multicenter study [20]. Second, care-dependent patients in our cohort had a higher burden of vascular risk factors that are associated with poor stroke outcome: they were older, had more severe stroke and more cardiovascular risk factors (e.g., arterial hypertension and diabetes mellitus) and up to one-third had already suffered from stroke before as compared to independent patients. These findings are in line with other studies reporting unbalanced baseline characteristics to the disadvantage of care-dependent stroke patients [15, 21, 22]. Third, dependent patients had less often a favorable outcome and a higher mortality risk as compared to independent patients (26.2% vs 44.4% and 46.4% vs. 25.5%, respectively). However, dependency status was not associated with clinical outcomes after adjusting for potential confounders. Thus, the unfavorable outcome in dependent patients is probably related to the differences at baseline (e.g., older age and stroke severity) and higher disease burden as mentioned above and not to the dependency status itself [23, 24]. It has been shown that the likelihood of favorable stroke outcome after EVT linearly decreases with age [25–27]. Furthermore, older patients with pre-stroke dependency may have a higher susceptibility for medical complications such as pneumonia and medical care may be more often withdrawn after thrombolysis according to patient’s preferences [15, 28]. Of note, the outcome of pre-dependent stroke patients without thrombolysis is worse in any case as untreated stroke patients with pre-existing dependency were reported to have a 2.2-fold higher mortality risk than independent patients [29–31]. Therefore, EVT should not be systematically withdrawn in care-dependent patients. Our results suggest a careful selection of care-dependent patients for EVT and an accurate adjusting of patient and family expectations with respect to the outcomes. Leker and colleagues reported that stroke patients with pre-existing disability treated with EVT may have a 4.4-fold increased risk for poor clinical outcome (mRS ≥ 4). However, the study size was rather small (23 dependent vs. 108 independent patients) and definition of outcomes different than in our study [32]. Goldhoorn and colleagues analyzed data of 157 dependent stroke patients from MR CLEAN registry and reported results that were very similar to our study [20]. Favorable outcome was seen in 27% of prestroke-dependent patients, compared with 42% of prestroke-independent patients (*p* < 0.05) [20]. After adjustment, prestroke dependency was not associated with less-favorable outcome, whereas intracranial bleeding risk was similar in both groups*.*[20] However, the authors defined 3-month favorable outcome as mRS 0–2 or not worsening of the mRS score [20]. In comparison, we routinely used a dichotomized classification of prestroke mRS either as 0–2 (independent) or 3–5 (care-dependent) rather than using a continuous numerical score for prestroke mRS. Thus, prestroke care-dependent patients with 3-month mRS 4 or 5 were not able to achieve favorable outcome (defined as mRS ≤ 3) in our study. We therefore assume that the rate of care-dependent stroke patients achieving favorable outcome after EVT may have been even higher in our study by applying the same outcome definition as reported from MR CLEAN trial [20]. Instead, we used a predefined sliding dichotomy analysis for favorable outcome (mRS 0–2 for independent patients vs. mRS 0–3 for care-dependent patients) as recommended for outcome assessment in unbalanced cohorts with varying prognostic factors [33]. Of note, literature on EVT in care-dependent stroke patients is sparse as care-dependent patients have been excluded from randomized controlled studies yet [34–37]. Karlinski and colleagues investigated 7250 stroke patients treated with IVT and reported that patients with prestroke dependency (mRS ≥ 3) were less likely to achieve favorable outcome at 3 months despite IVT (OR_adjusted_ 0.59; 95% CI 0.34–1.01; *p* = 0.055) [22]. In addition, dependency status was independently associated with mortality in both large IVT trials [15, 22]. In view of these results, one may speculate whether care-dependent patients with acute stroke might benefit more from EVT than IVT, but a firm conclusion is not possible due to lack of comparative studies. This is especially of relevance as dependent patients had a higher stroke severity (as measured by baseline NIHSS score) in our study as compared to the two abovementioned IVT trials, while EVT has been shown to be more effective treatment of severe stroke due to large vessel occlusion than IVT [5, 15, 22, 38]. Another interesting finding of our study is that the risk of sICH in care-dependent patients was not increased as compared to the counterpart (4.9% vs. 5%) although bleeding predictors such as older age, higher stroke severity, history of previous stroke and increased use of antithrombotics at stroke onset were distributed to the disadvantage of dependent patients [27, 39–43]. The risk of sICH in our cohort was also comparable to the bleeding risk in care-dependent patients treated with IVT (4.8%) and lower than in MR CLEAN registry (8%) [15, 44]. Thus, the unfavorable outcomes in dependent patients are not related to an excess of intracranial bleeding complications and EVT seems to be safe in care-dependent patients.

The main strength of this study is the high data quality due to systematic and prospective data collection at baseline and 3 months by certified neurologists. Assessment of the data, therefore, is unlikely to have been influenced by the current research question. Nevertheless, we are aware of several limitations.

First, this is a non-randomized observational study including a relatively small sample size of dependent stroke patients with unbalanced baseline characteristics in both cohorts. Second, a selection and treatment bias is likely in view of the observational study design and may not be completely removed through multivariate model. Thus, we urge to a cautious interpretation of our findings. Third, we were not able to assess the exact causes of pre-stroke dependency and the exact mRS score in dependent patients. Fourth, data were collected over 10 years during which incremental steps in stroke treatment were implemented. Especially recanalization techniques and acute stroke care treatment like stroke unit care and early rehabilitation might influence outcome in stroke patients. Nevertheless, EVT has a long tradition in our center and has been systematically performed. Finally, the rating of prestroke mRS might be challenging due to a high degree of interobserver variability [45, 46].

## Conclusion

This study revealed unfavorable outcomes in care-dependent stroke patients after EVT and suggests a careful patient selection for EVT. However, prestroke dependency should not be a reason to withhold EVT in these patients as outcome was rather related to unfavorable baseline differences in dependent patients (e.g., older age, higher stroke severity, history of previous stroke) and was not determined by the dependency status itself. Furthermore, EVT seems to be safe in care-dependent stroke patients with respect to similar risk of sICH in both cohorts. A well-powered randomized-controlled trial would be ideal to assess the safety and efficacy of EVT in care-dependent patients and to identify the patients who benefit most from EVT.

## Data Availability

Requests for access to the dataset from qualified researchers trained in human subject confidentiality protocols may be sent to Department of Neurology, University Hospital Berne (marcel.arnold@insel.ch).

## Code availability

R (version 3.1.2; R Core Team [2014]; R: A Language and Environment for Statistical Computing; R Foundation for Statistical Computing, Vienna, Austria).
